# A public health e-learning master’s programme with a focus on health workforce development targeting francophone Africa: the University of Geneva experience

**DOI:** 10.1186/s12960-015-0065-8

**Published:** 2015-08-13

**Authors:** Philippe Chastonay, Véronique Zesiger, Roberto Moretti, Marco Cremaschini, Rebecca Bailey, Erika Wheeler, Thomas Mattig, Djona Atchenemou Avocksouma, Emmanuel Kabengele Mpinga

**Affiliations:** Swiss School of Public Health Plus, Zurich, Switzerland; Institute of Global Health, Faculty of Medicine, University of Geneva, Geneva, Switzerland; Health Promotion Service ASL of Bergamo, Bergamo, Italy; IntraHealth International, Chapel Hill, NC USA; World Health Organization, WHO Geneva and Global Health Workforce Alliance, WHO, Geneva, Switzerland; Swiss Health Promotion Foundation, Berne, Switzerland; World Health Organization, WHO AFRO, Brazzaville, Congo

## Abstract

**Background:**

Shortage of a competent public health workforce is as a worldwide problem. The situation is especially bad in sub-Saharan Africa. In 2008, the World Health Organization and the Global Health Workforce Alliance launched a call for proposals for a public health training programme with an emphasis on health workforce development specifically targeting Africa. Our article presents the development, implementation and evaluation of an e-learning Master of Advanced Studies in Public Health on Workforce Development. The project was developed in collaboration with academic partner institutions of 10 French-speaking African countries and local/regional/HQ WHO offices.

**Methods:**

A five-step approach was adopted. First, a needs assessment study was done in the target countries, with identification of priority health issues. Second, student and tutor selection was done in collaboration with local WHO offices, health authorities and partner universities. Third, the e-platform was developed and a training workshop for tutors was organized. Fourth, the learning objectives were derived from the needs assessment study and an interactive educational approach was adopted. Fifth, the participation of students, their perception of the programme, their performance on assignments and community outcomes were monitored.

**Results:**

The needs assessment allowed the identification of 12 priority health issues (trauma related to road accidents, maternal and child health, HIV/AIDS, mental heath, food and malnutrition, health resource management, infectious diseases, access to essential drugs, chronic diseases, health promotion, ageing and violence/conflicts) of which 10 were studied through the lens of the key public health disciplines (epidemiology, human resources, health project/service planning, health policy, communication, health economics/management, informatics and ethics/human rights), each validated through a certifying examination. Student participation, measured through connection hits (total: 58 256; mean: 168/student/module) and posted messages (total: 5994; mean: 18/student/module), was good, and global satisfaction was high (7.7/10). Twenty-nine students out of 37 obtained their master’s degree from the University of Geneva. Outcomes reported include career development, strengthening of inter-country networks and common projects.

**Conclusions:**

Keys to the success of the programme were the enthusiasm and commitment of students, the availability of the coordination team, the simplicity of the electronic platform and the support of local/regional/WHO offices. Yet, the sustainability of the programme is not assured.

## Background

The World Health Organization has identified the shortage of competent health workforce as a major and worldwide problem [1]. Data from Africa show that the situation is especially bad in sub-Saharan Africa and that, though efforts have been made, more is needed [2]. One might especially mention the CARTA Initiative, which stands for the Consortium for Advanced Research Training in Africa. It is a south–south partnership with south–north collaboration having two main objectives: first, to strengthen research infrastructure and management capacity at African universities; second, to support doctoral training through a model collaborative PhD programme in population and public health. The Initiative has achieved some remarkable successes, having enrolled more than 100 fellows in its PhD programme supported by over 90 facilitators from 22 countries [3]. Yet, the situation remains critical in many countries further aggravated by an important brain drain [4]. Since out-of-country-training increases the risk of brain drain, new training methods especially via Internet technologies have been suggested [5]. Furthermore, in order to strengthen the public health workforce, core competencies of public health personnel have been proposed by international bodies, which may help solve health problems at various levels (regional, national, local) [6]. Yet, some authors have challenged the relevance of such globally defined core competencies and insist on local needs assessment prior to any training programme [7].

In 2008, the World Health Organization and the Global Health Workforce Alliance launched a call for proposals for a public health training programme with an emphasis on health workforce development specifically targeting Africa. Two proposals were accepted and funded in 2010, one from the University Western Cape (English) and one from the University of Geneva (French). The latter proposal had been developed in partnership with universities and ministries of health of 10 French-speaking African countries (Burkina Faso, Burundi, Cameroon, Chad, Central African Republic, Congo, DR Congo, Ivory Coast, Mali and Senegal) and in close collaboration with local/regional/HQ WHO offices [8, 9].

Our article presents the development, the implementation and the evaluation of the *Geneva e-module in Public Health GEMPH* – a distance-learning Master of Advanced Studies (MAS) in Public Health programme via an electronic platform coupled with specific workshops, based on a long-running residential MAS in Public Health of the University of Geneva [10].

## Methods

### Curriculum development, implementation and evaluation

Step 1: Needs assessment

- The project started with *a needs assessment* in spring 2010 through country visits (Senegal, Ivory Coast, Mali, Burkina Faso, Cameroon, Central African Republic, Congo, Democratic Republic of Congo) by one of the principal investigators (EKM). Chad was visited later on; the Central African Republic and Burundi were accepted at the request of WHO though they were not in the initial project. Needs assessment was done according to standard procedures, that is, through semi-structured interviews and focus groups with key informants in partner countries [11–13]. The needs assessment was complimented with a survey among the students (see below).

Step 2: Identification of country partner institutions, student selection and tutor selection, identification of country priority health issues and specific expected competencies

- During the above-mentioned country visits, local educational institutions as potential partners were identified: their possible role was discussed; furthermore, the support of health and education ministries was asked as well as the collaboration of WHO country offices.
- The project moved forward with the *student selection* in fall 2010. It was a multiple-step selection. First, WHO country offices and ministries of health gave a short list of potential candidates per country. The University of Geneva coordination team developed a test that the candidates took in fall 2010 including an essay on the public health situation of their country (identification of priority public health problems in their respective countries) and a self-evaluation of their public health competencies and training needs in public health [14]. The Geneva-based coordination team independently assessed the individual copies. The partner institutions proposed the local tutors.

Step 3: Tutor training and e-platform development (fall 2010)

- In fall 2010, an *electronic platform was adopted* upon the recommendations of the e-experts of the University of Geneva: the Moodle platform [15].
- In December 2010, *an educational workshop for local tutors* was organized in Bamako, Mali, with the aim to motivate local tutors and make them familiar with the project and its educational approach.

Step 4: Content development and educational approach (fall 2010 through spring 2013)

- The main learning objectives were derived from the above-mentioned training needs assessment and from the educational needs as identified by the candidates based on the priority health problems they were confronted with in their respective countries, taking also into account international recommendations.
- An *interactive educational approach* was adopted as recommended by a large body of educational experts regarding optimal adult learning strategies [16, 17].

Step 5: Programme implementation, monitoring and evaluation (winter 2011 through spring 2014)

- *Each priority health issue was treated over a 2-month period*, including reading assignments, exercises and group work.
- The *participation* of students, their *perception* of the programme, their *performance* on assignments (formative and certifying examinations) and possible community *outcomes* were monitored throughout the curriculum with various techniques (automatic monitoring of connection to the platform [15]; strengths, weaknesses, opportunities and threats (SWOT) technique evaluation [18]; learning objective achievement [19]; outcome measurements [20]).
- Students completed a final individual examination and a collaborative report on specific transnational health issues with a focus on health workforce development as a final topic.

## Results

### Step 1: Needs assessment (spring–summer 2010)

*Table*1*summarizes the major training needs as expressed by the key informants of the participating countries*. A hundred and six key informants (public health professionals and academics) of nine countries had been identified by the deans of the partner institutions and by the WHO country representatives: in nine focus group discussions, they identified major public health functions and tasks relevant for their respective countries. The detailed findings of the needs assessment study were published elsewhere [14].

**Table 1:** Identified training needs by key informants in partner countries

Public health function	Public health tasks
Planning/evaluation	Set public health objectives and priorities
	Develop public health projects
Leadership	Define public health strategies
	Define human resources policies
	Mobilize and coordinate resources
Coordination	Put results into practice
Research	Identify public health problems
	Collect public health data
	Draft public health research reports
Communication/information	Inform community and deciders on results
	Design and use appropriate information systems
Social marketing	Analyse the social and political environment
Negotiation	Be familiar with conflict management
Training	Ensure the provision of public health training
Management	Organize health services
	Manage public health archives
	Draft administrative reports

### Step 2: Identification of country partner institutions, student and tutor selection, country priority health issues and specific expected competencies (summer–fall 2010)

#### Identification of country partner institutions

Educational *institutions* agreed to participate in the project by helping students through logistic support and providing local tutors.^1^*The ministries of health and the ministries of education* of the partner countries also agreed to support the project, mainly in allowing some of their staff to take the programme. Furthermore, *the local WHO Offices*, with the support of the regional WHO office and the WHO HQ, played a key role in initially establishing a short list of potential students and in providing educational and logistic support during the implementation phase of the project (co-organizing the educational workshop for tutors, supporting logistically the selection process, co-organizing the final examinations). Eventually, the regional WHO office co-organized the final workshop at the end of the programme and the graduation ceremony.

#### Student selection and tutor selection

The coordination team reviewed the tests taken by the candidates according to a specific grid. Eventually, 34 students out of 55 candidates were selected with balanced country representation. In March 2011, three students from Burundi joined the programme upon the request from WHO increasing the total number of students to 37. Male/female ratio 3:1; 45% physicians, 24% managers and administrators and 31% health workers, teachers and social workers.

Each collaborating academic institution proposed a *local tutor* with an academic degree and proven competencies in public health ready to supervise the local students in their case studies: mainly senior public health academics were proposed.

#### Priority health issues

Priority health issues in the participating countries as identified/described by the candidates in their “selection” essays are listed below:

**Table:** 

- HIV/AIDS	- Infectious diseases	- Chronic diseases
- Mental heath	- Maternal and child health	- Food and malnutrition
- Trauma related to road accidents	- Access to essential drugs	- Health promotion
- Health resource management	- Ageing	- Violence/conflicts

#### Specific public health competencies

Specific public health competencies identified by the candidates as main learning objectives are listed in Table 2. Data were collected through a questionnaire addressing core competencies of public health professionals developed in the context of the planning and implementation of a master’s programme in public health at the University of Geneva [10] and have been published in extenso elsewhere [14], based on the recommendations of the WHO Educational handbook for health personnel [16] and the Council on Linkages between Academia and Public Health Practice [21].

**Table 2:** Learning objectives based on competency needs expressed by candidates of the programme

Each student should be able at the end of the programme to:
Implement health prevention and health promotion activities
- Develop health prevention and promotion strategies and action plans
- Implement health prevention and promotion programmes
- Support at the technical level health authorities
Collaborate and communicate
- Communicate with the population, with health authorities and with NGOs
- Collaborate with health professionals and coordinate common actions
- Council health authorities on the health of the population
Manage public health activities and structures
- Identify health priorities according to urgency and economic constraints
- Prepare public health projects including budget and legal aspects
- Analyse and formulate public health objectives (at local and national levels)
- Plan and manage health workforce development including life-long training
Develop and implement research activities
- Establish the health profile of the population at local and national levels
- Organize an information system to collect health data
- Analyse the financial impact of health promotion programmes
- Evaluate the efficacy and efficiency of public health programmes
- Design a public health research project
Train health personal
- Develop, implement and evaluate training programmes for health professionals
Self-evaluation
- Evaluate one’s activities in order to better perform

### Step 3: Tutor training and choice/development of the electronic platform (summer–fall 2010)

The *electronic platform* (Moodle) adopted allowed easy interactions between educators, learners and learning resources, offering asynchronous discussion forums and adaptability to the needs and demands of teachers and students. Furthermore, this choice gave access to the informatics back office of the University of Geneva, which supported the use of the platform throughout the programme and allowed smooth functioning.

An *educational workshop for local tutors* and WHO focal points was held in Bamako, Mali. The participants were academics and health managers (nine countries), WHO focal points (five countries), a representative of the Consortium RAS (one person), WHO Geneva representatives (two persons) and University of Geneva teachers (two representatives of the coordination team). The aim was to motivate local tutors and make them familiar with the project and its educational approach. It was also an opportunity to promote the programme locally and regionally and to initiate a network among local tutors and local/regional WHO representatives as well as the Geneva-based coordination team [22].

### Step 4: Content development and educational approach (fall 2010–fall 2012)

The *main learning objectives* were derived from the above-mentioned training needs assessment and from the educational needs as identified by the candidates based on the *priority health problems* they confronted within their respective countries (Table 2), taking also into account international recommendations [5].

As a consequence of the interactive educational approach adopted [16, 17], 10 priority health issues were identified: trauma related to traffic accidents, mother and child care, HIV/AIDS, mental health, food and malnutrition, human resources for health, infectious diseases, essential medicines, chronic diseases and health promotion. The 10 priority health issues were studied through the lens of the *key public health disciplines*: epidemiology, human resources, health project/service planning, health policy, communication, health economics and management, informatics and ethics and human rights. Ageing and violence/conflicts, two issues that had been identified, could not be treated as specific modules due to time and logistic constraints. Yet, considering their importance, ageing issues were mainly discussed during the modules mental health and non-communicable chronic diseases, whereas violence and conflict issues were mainly discussed during the modules HIV/AIDS, mental health and food–malnutrition. Specific educational tools were developed in order to explore the different priority health issues with focus on the acquisition of broad competencies in the various disciplines (readings, individual exercises, case studies, discussion forums, group work, community assignments with project implementation, data collection, data analyses, report writing, press release, etc.), based on tools developed in the MAS in public health of the University of Geneva [10].

### Step 5: Programme implementation, monitoring and evaluation (winter 2011 through spring 2014)

The programme was comprised of *ten 2-month modules* on the priority health issues identified, an individual *final certifying examination* and a *final group work*.

Each module started with required reading centred on a specific case study related to the identified priority health issue. Specific handouts, WHO documents, chapters of books and articles were made available on the electronic platform, and books in basic public health disciplines (epidemiology, planning health interventions, communication, ethics, human resource management) had been shipped to the students prior to the beginning of the programme. Further assignments included development of a collaborative glossary, specific exercises in several public health disciplines and development of *wikis*^2^ in order to promote collaborative work. Forum discussions on specific topics with compulsory participation were also initiated allowing exchange of experiences and some insight on country-specific issues. Depending on the health issue studied, specific individual or group work was required, such as writing of an essay, implementing a field investigation, planning a health intervention or collecting health data related to their own country or to a specific situation. At the end of each module, the students had to take a certifying examination of different types (Table 3). The final examination included a case study (management of the health situation of a refugee camp) with data analysis and the planning of an appropriate intervention (strategy–objectives–activities–resources allocation–evaluation) as well as the elaboration of a health prevention poster addressing the target population. Furthermore, students as a collaborative effort had to write an essay on human health workforce challenges in their countries.

**Table 3:** Students’ participation (connections to the electronic platform and messages sent)

Module	Total connections	Mean connections/student	Total messages	Mean messages/student
Trauma related to accidents	9756	279	741	21
Mother–child health	7998	228	896	25
AIDS/HIV	9435	269	995	28
Mental health	4811	137	371	10
Food and malnutrition I	5418	154	716	20
Human resources for health	5447	160	593	17
Infectious diseases	5530	162	792	23
Essential medicines	4198	123	306	9
Chronic diseases	3618	106	415	12
Health promotion	2045	60	169	6
Total/Mean	58 256	168	5994	18

First, students (in country groups) had to collect health data, and critically assess their quality, from their respective countries including data on the following:

- health indicators, such as life expectancy, mortality and morbidity rates;
- health services, such as the number of community health services, mental health institutions or intense health care units;
- the active human health workforce, such as the number of trained nurses, midwifes or physicians, but also more specific details such as wage practices, working hours, requirements of continued education or the carrier opportunities;
- the traditional medicine practices; and
- the role of international non-governmental organizations in the health sector.

Second, students (in inter-country groups) were assigned to write a 10- to 20-page essay using the collected data with the intention of editing a collective booklet.

This final assignment was only partially successfully achieved. In retrospect, the requirement was considered as too demanding by the coordination team. The decision was taken to validate the essays though they were not publishable at that stage.

The *participation of students* varied from one module to another: their connection frequency to the platform and messages posted are listed in Table 4; most connections were noted during the modules on “Accidents” and “HIV/AIDS”. The *students’ satisfaction* was measured on a 10-point scale (1: very unsatisfied; 10: very satisfied) for each discipline taught/studied and for each module. Globally, the satisfaction rate varied between 6.8 and 8.4 with a mean of 7.7. The highest ranking modules were the “Mental Health Module” and the “Essential Medicine Module”. Best considered taught/studied disciplines were “Informatics” and “Human Resources” (Table 4). Globally, as measured through a *SWOT* session during the final workshop [23], the coordination staff perceived as *strengths* notably the commitment and enthusiasm of students who managed to connect even in difficult local situations, while the students highly valued the exchange of experiences, the availability of documents on the electronic platform and the educational approach. *Weaknesses* mentioned by students were difficult Internet access (mentioned by 45 % of the students, forcing them to access via Internet cafés), limited to no involvement of local tutors (for roughly 80 % of students) (Table 5). The programme was also perceived as an *opportunity* for developing transnational collaboration and for strengthening the regional network.

**Table 4:** Students’ satisfaction according to module and discipline (1 to 10 scale: 1: very dissatisfied; 10: very satisfied)

Discipline	Mental health	Ess med	Hum res	Food	Inf dis	NCD	Hlth prom	Trauma/acc	HIV/AIDS	Mat and child	Mean
Informatics	8.6	8.4	8.4	8.1	8.4	8.3	8.4	7.8	7.8	7.0	8.1
Human resources	8.4	8.3	8.1	7.8	8.3	7.1	8.1	7.9	7.3	7.5	7.9
Epidemiology	8.2	7.9	7.8	7.9	7.8	7.8	7.8	7.4	7.9	7.5	7.8
Health policy	8.3	7.9	8.1	7.9	7.5	/	/	7.8	7.5	7.4	7.8
Health econ and management	8.1	8.1	7.9	8.0	7.6	/	/	7.5	7.4	7.3	7.7
Communication	7.8	7.4	8.0	7.3	7.4	8.0	7.3	7.8	7.4	7.0	7.6
Planning	7.9	7.7	7.3	7.6	7.8	7.5	7.3	7.3	7.6	7.0	7.5
Ethics and human rights	8.3	7.2	7.2	7.4	7.4	7.3	7.3	7.0	6.9	6.8	7.3
Mean	8.2	7.9	7.8	7.8	7.8	7.7	7.7	7.6	7.5	7.2	7.7

**Table 5:** Global evaluation of the programme (SWOT technique at the final workshop with students and teaching staff)

**Successes**	**Weaknesses**
Rapid feedback on assignments handed in	Globally non-functioning local tutorship with little local support (to students)
Exchange of experiences among students (andragogy approach)	Difficulties with Internet connections
Availability of relevant documents on the platform	No electronic material support (no computers offered to students by the programme coordination)
High participation rates and enthusiasm of students	Suppression of two modules initially planned (ageing and violence)
Low dropout rate (3/37) despite some political instability and limited Internet access	Difficulties to implement well-functioning group work, especially “writing a scientific report together”
Increase of UNIGE visibility at country levels in French-speaking Africa	Low student participation at the wiki assignment
Reliability of the Internet platform and the UNIGE-based IT back office	
**Opportunities**	**Threats**
Development of local and regional collaborations and networking	Financial sustainability
Use of new Internet technology as a training support	Local political insecurity
Possibility to extend the programme to other contexts	Plagiarism
	Workload for coordination and teaching staff

Twenty-nine students out of 37 achieved the *educational objectives*. Three students dropped out due to the unstable local political situation in their country, and five failed at least two modules and the final examination. The 29 students who fulfilled all programme requirements obtained a Master in Advanced Studies in Public Health from the University of Geneva with focus on Health Workforce Development (MPH equivalent free of charge, which might have provided an incentive for students to complete the course).

Noteworthy job performance outcomes included the following.

- The weakly use of newly acquired competencies during the programme is reported by two thirds of the students.
- Several students (12) achieved a professional promotion, mostly at ministries of health, in the year following their graduation.
- Several projects (6) developed throughout the course had been initiated and implemented locally at the time of this publication.
- A collaborative network among students has been established and is functioning on a regional basis.

## Discussion

A shortage of competent clinical and public health professionals, with knowledge and skills in health workforce development, has been a long-standing issue in most of sub-Saharan Africa, bearing 25 % of the global burden of disease and having only 3 % of the health workforce [1].This was the driving factor behind the development of an e-learning master’s programme with focus on health workforce development coordinated by the University of Geneva with technical and financial support of the World Health Organization and the Global Health Workforce Alliance [8].

An initial assessment of needed public health competencies was completed through a survey of 106 representatives of health authorities of the participating countries (Table 1) [14]. The initial survey was complemented by an assessment of expressed training needs among the candidates during the selection process. This allowed defining more precisely learning objectives relevant to the students’ training expectations/needs (Table 2), as recommended in the literature [16, 24]; it proved useful in our case because it appeared to be very motivating.

The chosen distance-learning approach, while part of the call for proposals, was also an opportunity to test Ruiz’s statement, “the integration of e-learning into medical education can catalyze the shift toward applying adult learning theory, where educators will no longer serve mainly as the distributors of content, but will become more involved as facilitators of learning and assessors of competency” [25]: indeed, the reality of connectivity problems in many of the partner countries kept us from seriously considering real-time synchronous lectures as a viable teaching model. Therefore, asynchronous case studies with online readings, exercises, chats and wikis around priority health issues appeared as the only realistic teaching modality, which in turn allowed interactivity among students, course facilitators, collaborative work and exchange of experiences, thus being in tune with modern adult learning strategy recommendations [16, 17].

The e-learning approach also made the programme available to candidates that could not have taken the residential course at the University of Geneva [10]: this is certainly one of the advantages of distance-learning programmes among a long list of possible benefits if principles of effective learning are integrated [26], which was done as mentioned above. Furthermore, the priority-health-issue approach with case studies relating to realities of the working and community environments of students contributed to the motivation of students and was certainly an asset of the programme design, though motivation might also have been influenced by the perspective of a free-of-charge diploma. The local tutoring, which seemed initially as an important feature of the programme, also advocated by many authors [25, 27], neither responded to the students’ nor to the coordination team’s expectations, though a specific workshop had been organized for the local tutors [22]: close-distance tutoring from the Geneva-based coordination team became therefore a major activity. A lack of availability of local tutors was put forward by the students, mainly related to professional obligations of tutors who were senior academics/professionals with international mandates. Midlevel professionals more readily accessible to students yet competent in the field of public health might have been a better choice, as experienced in another distance-learning programme of the University of Geneva [28]. Yet, more drastic measures might be necessary to improve the commitment of local tutors, such as a clear decision of the University authorities putting tutorship high on the academic agenda, which at present is not the most frequent situation [29].

The selection of students was a complex procedure. It was greatly facilitated through the close collaboration of the WHO HQ, regional and country offices. Being a competitive, multiple-step procedure, it eventually allowed selecting of students as a self-assessment process and a personal essay showing their knowledge of public health problems and challenges their respective country of origin were facing, thus insuring a certain degree of “knowledge homogeneity”, as recommended by some authors [30]. It further allowed reducing the risk of favouritism and nepotism which make a rather common problem in such contexts [31], as the final decision of acceptance was the sole responsibility of the Geneva-based coordination team. The dropout rate was low (<10 %): similar dropout rates have been reported in the literature for degree-granting distance-learning courses in sub-Saharan Africa, while dropout rates up to 70 % are mentioned for MOOCs [32].

The electronic platform Moodle was easy handling for the teaching staff, benefited from the IT back office of the University of Geneva, and did not represent a major difficulty for most of the students. The difficulty for students was to connect from their office, and several had to connect via the local Internet Cafés at their expense. The Moodle platform also allowed many different educational approaches as mentioned above, which is also reported as one of its strengths in the literature [33].

The systematic monitoring of the programme allowed measuring student participation and satisfaction as mentioned in the “Results” section. The participation estimated via log-ins and messages posted varied from one module to another, with maximal interest shown for modules “Trauma post Accidents”, “HIV/AIDS” and “Mother and Child health” followed by “Infectious Diseases” and “Food/Malnutrition”, issues that rank high among health priorities in sub-Saharan Africa as reported in the Kaiser/Pew Global Health Survey [34]. The globally high participation rate of most students despite sometimes difficult local situations might also be related to the observed team spirit that emerged over the months among “building sense of community at distance” as some authors have stated [35]. Satisfaction of students as measured on a 10-point scale (10: very satisfied) lies in the mean between 7 and 8 for all the modules and all the disciplines: this can be considered as a high approval rate, which in turn might have some impact on the motivation and performance of students [36]. Yet, one might consider that students’ satisfaction evaluation is of limited relevance when done at the end of the teaching sessions: it could be more informative after the students had the opportunity to put their learning into practice as suggested in a study among alumni of a MPH programme [37].

For each module, one or two certifying examinations had to be taken by each student before they could move to the next module: these examinations tested the knowledge and competencies meant to be acquired during the module (Table 6). Furthermore, a final exam tested in an integrative way, as recommended by some authors [16], the newly acquired knowledge and competencies. Eventually, 78 % of the students succeeded and qualified for the master degree.

**Table 6:** Type of certifying examination of each module

Module	Exam 1	Exam 2
Trauma related to accidents	Participation	–
Mother–child health	Epidemiology test	Production of a glossary (group work)
AIDS/HIV	Epidemiology test	Planning of a public health intervention
Mental health	Qualitative survey	Writing a report on the qualitative survey
Food and malnutrition	Epidemiology test	Planning of a public health intervention
Human resources for health	Presentation of websites on human resources for health	Identification of hospital human resources economic indicators
Infectious diseases	Planning of a public health intervention	Communication (prevention campaign)
Essential medicines	Epidemiology test	–
Chronic diseases	Data analysis and interpretation	Evaluation report of a local health project
Health promotion	Epidemiology test	Evaluation report of a local health project

The global evaluation (Table 3) of the programme highlighted strengths such as “exchange of experiences among students”, “quality of the feedback on assignments” and “commitment of students despite difficult local situations”. It also highlighted weaknesses, such as little support from local tutors, difficulties in access to Internet and little material support. Development of collaborations and strengthening of networks were considered as opportunities, while lack of long-lasting financial support of the master’s programme and local political instability were considered as principal threats to the programme, and indeed, we experienced three dropouts because of local insecurity/war, a phenomenon reported by others [38]. These observations, not very different from evaluation results of online, distance-learning programmes reported in the literature [39, 40], gave valuable information in order to adapt the curriculum and improve the programme: integrating monitoring and evaluation mechanisms early on the planning of any training programme, as recommended [41, 42], should be a priority to any curriculum designer.

Observed outcomes such as the use of newly acquired skills in professional life, career development and initiated collaborative projects suggest that the master’s programme had some impact “beyond the classroom”, which should ultimately be the aim of any training programme in public health [43].

## Conclusion

### Summing up: what are the lessons learned?

First, interactive training methods integrating the students’ experience and their professional/community realities contributed to the motivation of students and set the basis for collaborative work among them.

Second, though the Internet platform was easy to use and no major malfunction occurred, there were local connection difficulties, which had some negative impact keeping at times the programme from smoothly functioning.

Third, local tutoring did not respond to expectations, perhaps because the tutors selected were overbooked senior public health academics. Careful attention should be given to defining terms of reference of tutors, the criteria for their selection and incentives to encourage performance. But above all, such programmes should be identified as priorities in the strategic plans of the collaborating universities, thus allowing to reward in academic terms (career) serious commitment of tutors in such programmes.

Fourth, long-term financial support could so far not be ensured, which might compromise the sustainability of the programme, though partner institutions would welcome it, despite several financial requests to various agencies working in development and international cooperation. Perhaps a different financing approach should be considered, that is, a financing mechanism based on funding scholarships to students rather than funding the teaching institution. One might also argue that international organizations should take the leadership in funding such programmes in close collaboration with higher education institutions.

Fifth, the master’s programme was a very enriching experience for the University of Geneva, especially for the coordination team of the project, who is at present developing public health projects with partner institutions and former students. It also indirectly contributed to a strategic decision at the University of Geneva: the development of an African Studies track.

## Endnotes

^1^List of partner institutions : Unité de Formation et Recherche en Sciences Médicales, Université de Bouaké, Abidjan, Côte d’Ivoire; Faculté des Sciences de la Santé, Université Marien NGouabi, Congo Brazzaville; Faculté de Médecine, Université de Mbuji-Mayi, République Démocratique du Congo; Faculté des Sciences de la santé, Université de Ndjaména, Tchad; Faculté de Médecine, Pharmacie et Odontostomatologie, Université de Bamako, Mali; Faculté de Médecine, Université Cheik Anta Diop, Dakar, Sénégal; Unité de Formation et Recherche en Sciences de la santé, Université de Ougadougou, Burkina Faso; Faculté des sciences biomédicales, Yaoundé, Cameroun; Faculté des Sciences de la santé, Université de Bangui, Centre Afrique

^2^A *wiki* is a collection of collaboratively authored web documents. Basically, a wiki page is a web page everyone in a class can create together with his/her colleagues. Consensus emerges from the work of the students themselves. *Wikis* can be a powerful tool for collaborative work: an entire class can edit a document together, creating a class product, or each student can have his/her own *wiki* and occasionally share it with classmates.
